# The Historical Significance of Titicut Follies in Psychiatric Treatment: An Anti-Censorship Perspective

**DOI:** 10.1177/00332941211061080

**Published:** 2022-01-31

**Authors:** Salvatore B. Durante

**Affiliations:** Department of Educational Psychology, 3158 University of Alberta, Edmonton, AB, Canada; Forensic Psychiatry, Alberta Hospital Edmonton, Edmonton, AB, Canada

**Keywords:** ban, censorship, obscene, psychiatry, unintended consequences

## Abstract

Works of art and information judged as obscene can be censored or banned. This brief review evaluates the costs and benefits of censorship and the banning of artwork and information. In the history of psychology, Frederick Wiseman’s film *Titicut Follies* epitomizes the disadvantages of concealing art content. Despite protecting the privacy of patients, the ban of *Titicut Follies* delayed the reform of psychiatric treatment practices and hospitals. The decision to censor or ban artistic and scientific information can result in the loss of knowledge and potential improvements to social, political, and economic institution practices.

Technological advances in the 21st century have allowed people easy access to a vast amount of artistic and scientific content. Smartphones, applications, and social media services (e.g., Facebook, Instagram, TikTok, and Twitter) have transformed how people consume information and forms of art, connect with individuals, and convey their knowledge and lived experiences. Despite the benefits of improved worldwide connectivity and quick access to a wide range of information, there are growing concerns with the rise of people creating content that distorts facts and reality (Bakir & McStay, 2018; Pennycook et al., 2020a, 2020b; Pennycook & Rand, 2020). For example, COVID-19 misinformation has led some people to use ineffective home remedies or disregard the virus entirely (Pennycook et al., 2020b). Furthermore, the spread of fake news and propaganda pictures and videos during the 2016 and 2020 United States presidential elections caused political, racial, and social division that culminated in the Capitol building insurrection on January 6th, 2021 (Bak-Coleman et al., 2021; Bakir & McStay, 2018; Pennycook et al., 2021; Simon, 2021).

Misinformation and distortions of reality through diverse modes of artistic expression or media (e.g., newspapers) are not limited to the 21st century. D. W. Griffith’s (1915)*The Birth of a Nation* created many cinematic innovations. However, its narrative was sympathetic to the Ku Klux Klan’s racist ideology and brutality towards African Americans (Chiang & Posner, 2006). Consequently, *The Birth of a Nation* revived and strengthened the Ku Klux Klan’s power in the United States. Similarly, Adolf Hitler and his Nazi regime created numerous anti-Semitic conspiracy theories that were radio broadcasted and presented in newsreels, films (e.g., *The Eternal Jew* [Hippler, 1940]), posters, and newspapers. The result of Nazi propaganda was the persecution and genocide of the Jewish people.

An approach to manage past and present inaccurate information and offensive artwork or perspectives is to apply warning labels to inform people that the content is false or potentially upsetting (Pennycook et al., 2020a). Pennycook, Bear, Collins, and Rand conducted a study that evaluated the impact of warnings on people’s beliefs about the accuracy of the information and their willingness to share the content. In Pennycook and colleagues’ study, participants were randomly assigned to one of two conditions. In the first condition, participants were presented 12 true and 12 false news headlines without any warnings. In the second condition, participants were shown six false news headlines with warnings, and six false and 12 true headlines without warnings. Overall, Pennycook and colleagues found that when false news headlines had no content warning labels, participants were likely to view the content as accurate (i.e., an implied truth effect) and were more willing to share the headlines with others over social media. Therefore, using warning labels on social media posts can be challenging because verification errors can lead to false information being perceived and shared as fact. Regarding the impact of warning labels or media rating systems within the arts and people’s grading of their attraction towards artistic content, several studies have provided mixed results (i.e., warning labels or media rating systems can promote, repel, or have no effect on people’s interest in a particular artwork; Bushman & Cantor, 2003; Christenson, 1992; Deflem, 2020; Gosselt et al., 2012). Furthermore, it appears that trigger warnings (i.e., statements before disturbing content) have minimal effects on avoiding and alleviating discomfort (Jones et al., 2020; Sanson et al., 2019).

An alternative approach to warning people about content is the utilization of censorship which involves changing (e.g., replacing or removing profanities in a book, film, or television show) or banning content deemed obscene. The rationale to censor content is related to the political, religious, and social context for a nation (i.e., moral, government, political, or religious reasons lead to censorship of scientific information and artwork; Ahmad, 2019; Dunkel & Hillard, 2014). For example, Twitter, Facebook, and other social media platforms ban posts that are misleading or promote hate and violence towards people based on (but not limited to) their age, culture, gender, race, religion, and sexual orientation. However, in some instances, an outcome of social media censorship is that it can transition people to controversial online platforms (e.g., Parler and 8kun) where there are no limits on freedom of expression and extremist views and disturbing content are easily shared. Nevertheless, the effect of censorship of artwork can be abstract (Maliţa, 2019; Yan, 2017).

Arguments favoring censorship often emphasize that editing or banning information or artwork protects people from violent and disturbing content (e.g., child pornography and snuff films) and avoids individuals spreading false or hateful written or spoken matter (Maliţa, 2019; Yan, 2017). Censorship, therefore, puts limits and occasionally negates people’s freedom of expression rights by subjecting art content and information to an obscenity screening process (Dunkel & Hillard, 2014; Godfrey, 2007; Stewart, 2020; Yan, 2017). For example, in the United States, the Miller test (see Miller v. California, 1973) has been used for labeling information or artwork as obscene or appropriate for the general public (Stewart, 2020). The Miller test evaluates obscenity by examining if the content evokes excessive and appeals to sexual interests, depicts or describes in an overtly offensive way sexual interactions, and lacks artistic value (Stewart, 2020, p. 1256). Currently, an agreed-upon universal definition of obscene is hard to achieve in legal and social contexts (Godfrey, 2007). Potentially, an issue with defining obscenity is that individuals in positions of power (e.g., judges, social media content moderators, and elected officials) may lack the knowledge base to distinguish between decent versus obscene content and overly rely upon unreliable and invalid measures of obscenity (Stewart, 2020).

Rather than allowing individuals to utilize critical thinking and discretion, obscenity laws aim to hinder exposure to art content under the justification that the artwork will foreseeably cause harms to psychological and physical health (Yan, 2017). Given the law’s reliance on indirect or assumed harms to peoples’ wellbeing, the continued maintenance of obscenity laws in many countries’ legislations is insubstantial. For example, criminal laws that describe direct links to overt and covert injuries to psychological or physical wellbeing are already in place (i.e., laws related to sex and child pornography, sexual assault, conspiracy, defamation, and murder; Maliţa, 2019; Yan, 2017). Therefore, obscenity laws are redundant legislation; and the subsequent use of censorship as a consequence of subjective categorization of information or artwork as indecent is a violation of freedom of expression under the claim of implied malicious intent (i.e., *mens rea* or guilty mind).

A problem arising with censorship is that it is finite, and, as a result, censored or banned content is frequently re-considered and embraced for its artistic and social value (Yan, 2017). For example, *Borat* (Charles, 2006) was initially denounced and banned in Kazakhstan (Yeung, 2020). However, upon the release of *Borat Subsequent Moviefilm* (Woliner, 2020), Kazakhstan embraced the Borat character and used his catchphrase (“very nice”) in various tourism advertisements (Yeung, 2020). According to Yan, censorship can impede political and social reform by silencing people challenging and representing social issues (e.g., violence, economic inequalities, racism, and misogyny) through various modes of artistic expression.

Given that censorship can delay reform, the purpose of this paper is to examine how the ban of Wiseman’s (1967) documentary, *Titicut Follies*, slowed progress in psychiatric treatment. This paper takes an anti-censorship perspective because of the issues related to obscenity laws, the mixed evidence for the effectiveness of censorship in preventing injuries to psychological and physical health, and the hindrance to social reform. The paper discusses censorship in film and television representations of mental health issues. This paper examines the social climate during the 1960s with today’s context; and provides a synopsis of *Titicut Follies* paired with an exploration of the ban of *Titicut Follies* on psychiatric treatment reform. This article also includes recommendations about how films like *Titicut Follies* can be used for anti-stigma training for health care providers in mental health settings and concludes with a discussion about censorship and the associated social consequences.

## Censorship in Film and Television Representations of Mental Health Issues

In an evaluation of film depictions of mental health issues, Moylan et al. (2020) conducted a 50-year review of films (i.e., films from 1960 to 2010) to determine which films accurately represented the social context of psychiatric treatment. Films such as *Titicut Follies* (Wiseman, 1967) and *One Flew Over the Cuckoo’s Nest* (Forman, 1975) were identified as representative of the deinstitutionalization movement (see Goffman, 1961); and providing non-fictional and fictional depictions of the inhumane treatment of individuals with mental health concerns and the authoritarian approach of some 1960s and 1970s psychiatric staff. Moreover, films such as *Rain Man* (Levinson, 1988) and *Girl, Interrupted* (Mangold, 1999) portrayed the lack of patient autonomy in psychiatric treatment and the emerging humanistic and behaviorist treatment approaches in psychiatric care.

Concerning the representations of mental health and psychiatric treatment on television, *The Sopranos* (Chase et al., 1999–2007) provided storylines that brought attention to the ethical and treatment complexities related to individual psychotherapy and utilization of psychotropic drugs. Alternatively, the documentary television series, *Couples Therapy* (Kriegman et al., 2019) records the everyday issues faced by couples in psychotherapy. In addition, *Couples Therapy* documents Dr. Orna Guralnik’s insights about her clients and provides viewers an understanding of the impact of psychotherapy on her wellbeing. Furthermore, the HBO series *Euphoria* (Levinson et al., 2019) was lauded for its accurate depiction of substance use and mental illness during adolescence (Kaufman et al., 2021).

Not surprisingly, film and television shows are modes of artistic expression that can influence how the public understands individuals with mental health concerns and psychiatric institutions (Moylan et al., 2020). For example, Durham and Wilkinson (2020) observe that films like *Joker* (Phillips, 2019) reinforce stigma towards individuals with mental health concerns by presenting fictional narratives that suggest that people with mental health issues are more prone to violence. Concerning television, Kaufman (2011) explains that some mental health professionals have expressed concerns with reality television shows and documentaries recording people with substance-related and addictive disorders (e.g., *Intervention* [Benz and Branton, 2005–2021], *Celebrity Rehab with Dr. Drew* [Pinsky and Irwin, 2008–2012], and HBO’s *Addiction* [Nevins, 2007]). The concerns expressed by mental health professionals are that reality television shows and documentaries about addiction are exploiting vulnerable populations for voyeuristic purposes rather than ethical psychiatric treatment or improved mental health awareness initiatives (Kaufman (2011)).

The censorship of films and television shows depicting topics in mental health has occurred through limits on what can be said and shown (e.g., violence, sex, smoking, and alcohol and drug consumption). A contentious issue in depicting mental health concerns is the portrayal of suicide (Cruikshank & Sevigny, 2020; Till et al., 2010). For example, Till and colleagues evaluated the emotional and mental states of non-suicidal participants who viewed films depicting suicide. After film watching, Till and colleagues measured the emotional and mental states of participants with various questionnaires. Till et al. found that participants reported being sadder post-film viewing. However, participants also indicated that they felt better about themselves. Till et al. suggest that participants improved emotional and mental state was potentially due to individuals comparing their lives to the films’ protagonists. Also, Till and colleagues’ results provided some evidence that censoring scenes depicting suicide did not alleviate negative emotional or mental states. However, Till et al. note that they could not conclude that editing scenes did not influence participant wellbeing. In a literature review examining the impact of media representations of suicide and subsequent suicides (i.e., suicide contagion), Domaradzki (2021) found that there can be a Werther effect (i.e., individuals are influenced by the depiction of suicide and consequently, imitate suicidal behavior and/or die by suicide), a Papageno effect (i.e., individuals exposed to a portrayal of suicide find non-suicidal solutions to their life concerns), or no effect on people.

Despite the mixed findings in regards to media reports and portrayals of suicide in film and television and the potential suicide contagion, mental health professionals and governing bodies (e.g., the World Health Organization) have developed guidelines around how to report or represent suicide as a way to prevent iatrogenic consequences to people’s mental health and to preserve life (Cruikshank & Sevigny, 2020). The presence of guidelines, therefore, forces people involved in film and television to confront their freedom of expressions rights and their moral obligation (i.e., beneficence and avoidance of direct or indirect harms to people’s mental and physical health) to the public in the creation of art content (Scalvini, 2020).

The limits of freedom of expression in the portrayal of suicide came to the forefront in 2017 when Netflix released the television show *13 Reasons Why* (Gomez et al., 2017-2020). The television show portrays the life and death of an adolescent girl named Hannah Baker who leaves a series of tapes explaining her motives for ending her life. In the depiction of suicide, *13 Reasons Why* includes a graphic scene showing Hannah’s suicide by cutting her wrists in a bathtub. The inclusion of Hannah’s death scene was met with much public scrutiny from the mental health and suicide prevention community who affirmed that the show glamorized suicide and would lead to an increase in adolescent suicides (Arendt & Romer, 2020; Copeland, 2020; Cruikshank & Sevigny, 2020; Ferguson, 2019, 2021; Scalvini, 2020). As a result of the criticism, Netflix decided to remove Hannah’s death scene in 2019.

Given the assertion that *13 Reasons Why* increased adolescent suicide, a correlation study conducted in 2019 (Niederkrotenthaler et al., 2019), and a time series analysis published in 2020 (Bridge et al., 2020), concluded that the release of the show was associated with an increase in adolescent suicides. However, Ferguson (2021) found that the association studies and, specifically, Bridge et al.’s study had inconsistent results as there was an increasing number of adolescent males ending their lives before the release of *13 Reasons Why* (NB. Bridge et al. suggest this is due to the promotion of the show). Also, Ferguson (2021) argues that results from the association studies were mixed because adolescent male suicides increased while adolescent female suicides decreased post-release of *13 Reasons Why*. Regarding this finding, the suicide contagion is questionable given that typically more female than male adolescents would end their lives because of the show’s female protagonist’s death by suicide. Overall, the inconsistent results in studies evaluating teen suicide during the release of *13 Reasons Why* could be due to seasonal and yearly changes in suicide rates amongst adolescents (Ferguson, 2021) or underlying factors influencing the variance in viewer responses to portrayals of suicide (Arendt & Romer, 2020). Therefore, people making public statements about the impact of the depictions of suicide on the rate of people ending their lives by suicide should be cautious because insufficient data is justifying the censorship of art content with portrayals of suicide (Ferguson, 2021; Scalvini, 2020).

Generally, film and television shows about individuals with mental health concerns and psychiatric institutions are rarely banned or made inaccessible. More often than not, films and television shows are edited to appease rating boards or avoid offending the general public audience. Taking this into account, the ban of Wiseman’s *Titicut Follies* is a rare outcome, and the consequences of this ban are overlooked and, at times, forgotten in the history of psychiatric treatment (Szasz, 2007).

## Titicut Follies

In 2017, *Titicut Follies* was turned into a ballet for audiences interested in an interpretation of the psychiatric treatment conditions of the 1960s that Wiseman depicted in his documentary (Cooper, 2017). Modern audiences evaluating the social climate of the 1960s may draw parallels to the political and social unrest occurring in the late 2010s and early 2020s (e.g., the civil rights movement and the movement for Black Lives, the Vietnam War, and the Iraq War). Concerning psychiatric treatment in the 1960s, individuals such as Thomas Szasz, R. D. Laing, Erving Goffman, and Michel Foucault influenced the emerging anti-psychiatry movement that questioned how people with mental health concerns were diagnosed and the treatment approaches used to alleviate their psychological and interpersonal functioning issues (Williams_and_Caplan_2012). Also, in the United States, President John F. Kennedy signed the *Community Mental Health Act of 1963*, which aimed to reform the national mental health system and implement humanistic approaches to psychiatric care (Erickson, 2021).

*Titicut Follies* is Wiseman’s observation (without narration or commentary) of the daily activities of the staff and patients/inmates at Bridgewater State Hospital for the Criminally Insane (see Grant, 1998; O’Rawe, 2019; Szasz, 2007). The title of the documentary is derived from the talent show put on by the hospital staff that is shown at intervals throughout the film. Despite categorization in the cinema verite style of documentary filmmaking, Wiseman prefers to describe his documentary filmmaking style as reality fiction (i.e., non-objective and a constructivist depiction of reality through film). With this filmmaking style, Wiseman presents various scenes of inhumane treatment of patients/inmates and staff indifference to and teasing of patients/inmates. For example, a patient/inmate named Jim is shaved haphazardly by staff who openly mock and agitate him for their amusement.

Wiseman draws attention to problems in the treatment approach for individuals with mental health concerns at Bridgewater State Hospital. The film’s audience views a psychiatrist’s disregard for the complaints of a patient/inmate named Vladimir who avers that he is better and blames the institutional environment as the cause of his mental health distress. The response by the psychiatrist and staff to Vladimir’s beliefs is an increase in his medication dosage and a diagnosis of schizophrenia. In addition, the film audience witnesses another patient/inmate named Malinowski (who has avoided eating for three days) being forced fed by his psychiatrist and the hospital staff. Wiseman utilizes parallel editing during the forced feeding scene to exhibit the funeral preparation process for Malinowski.

*Titicut Follies* was shown at the 1967 New York Film Festival as part of the “Social Change in America” program and had a six-day commercial run (Grant, 1998). The documentary received multiple poor reviews from critics as some expressed that the film violated decency and had no reason to exist beyond legislative and non-artistic consequences (Grant, 1998). Public screenings of *Titicut Follies* were banned by the Massachusetts Supreme Court, and Wiseman was involved in multiple court cases. In 1991, the ban was lifted by the courts given that First Amendment rights were given precedence over patient/inmate privacy rights (NB. there were no further court cases related to the film post-1991). Subsequently, in 1992, *Titicut Follies* aired on the Public Broadcasting Service (PBS) for general audiences (Moylan et al., 2020; O’Rawe, 2019; Szasz, 2007). The legal battles and ban of *Titicut Follies* were mainly due to concerns that the film was insensitive, grotesque, violated patients/inmates’ privacy and dignity, and there were discrepancies with whether the state government or Wiseman had final edit control. Despite the legal battles and ban, *Titicut Follies* was eventually allowed to be shown as an educational resource in the 1980s for mental health professionals (e.g., psychiatrists, psychologists, and psychiatric nurses) and students and other professionals in human services (Moylan et al., 2020). Nevertheless, the limited viewing audience did not lead to calls for changes at Bridgewater State Hospital, and in 1987, five patients/inmates died, and three of them died by suicide (Grant, 1998).

## Discussion

On August 25th, 1987, Wiseman was interviewed on *Nightline* by Ted Koppel who was doing a story on Bridgewater State Hospital (Grant, 1998). In the interview, Koppel questioned why Wiseman was against a censored screening of *Titicut Follies* which led Wiseman to say the following:

The censoring of *Titicut Follies* or any other film prevents people in a democracy from access to information which they might like to have in order to make up their minds about what kind of society they would like to live in—it is as simple as that. (Wiseman as cited in Grant, 1998, p. 250)

The effects of an edited version of *Titicut Follies* on mental health professionals and the general public are unknown. However, Wiseman’s comment confronts how censorship dilutes an artist’s representation of peoples’ lived experiences and aspects of the social milieu. Furthermore, Wiseman’s response suggests that censorship removes the possibility for film and other artworks to act as catalysts for political and social reform (Yan, 2017).

Given the momentum of the anti-psychiatry movement and the issues with the inhumane treatment of individuals with mental health concerns in hospital and forensic settings, it is possible that a wide distribution of *Titicut Follie*s in 1967 could have led to public outrage and reform of psychiatric treatment and institutions. This outcome is plausible given that when *Titicut Follies* was released and subsequently banned and entangled in numerous court cases, many American psychiatric institutions were closed or forced to change their treatment practices as a result of court orders (Goodman, 1993). The closure and changes occurring at psychiatric institutions were conceivably due to the legal consequences associated with the film’s ban and Wiseman’s depiction of the apathetic behavior of the hospital staff towards patients/inmates that were visibly in distress (Grant, 1998; Moylan et al., 2020; O’Rawe, 2019; Szasz, 2007). Regardless, the ban of *Titicut Follies* maintained inhumane treatment and indirectly allowed for the deaths of patients/inmates at Bridgewater State Hospital and other mental health institutions. The ban, therefore, impeded the potential for systemic reform of psychiatric treatment and institutions (Szasz, 2007).

In 21st century psychiatric care, *Titicut Follies* is used in the education of health care providers in mental health settings (Moylan et al., 2020). The utilization of films like *Titicut Follies* as educational resources may bolster programs to promote the anti-stigma of individuals with mental health concerns (Altindag et al., 2006; Carrara et al., 2020; McCann & Huntley-Moore, 2016). It is recommended that uncensored versions of films depicting topics in mental health be presented to professionals and students in mental health settings. Uncensored versions of films can allow mental health professionals and students to apply their critical thinking skills to evaluate the accuracy of the depiction of mental illness and psychiatric treatment and to compare their clinical experiences to the film content. Potentially, films about mental health issues and experiences with individuals with psychological and interpersonal functioning issues can increase mental health providers’ and students’ knowledge about psychiatric topics and facilitate anti-stigma attitudes towards individuals with psychological and interpersonal functioning concerns (Carrara et al., 2020). *Titicut Follies* can also be used in mental health training programs as an example of authoritarian psychiatric practices that must be avoided given the iatrogenic consequences to people’s mental and physical health.

Beyond application in mental health settings, *Titicut Follies* can be made more accessible on various movie and television streaming services to stimulate anti-stigma initiatives for and empathy towards individuals with mental health concerns in the general public. Alternatively, *Titicut Follies* on streaming services can act as a reminder to audiences of a period where dehumanization and unethical treatment of individuals with mental illnesses was the norm, and therefore, a point in history that must be acknowledged (without censorship) and never repeated.

Despite Wiseman being tactless in his portrayal of inhumane treatment (Grant, 1998), the ban of *Titicut Follies* removed any possibility for protests against and immediate calls for reform of the unethical and inhumane treatments at Bridgewater State Hospital and other psychiatric hospitals. Wiseman captured on film a disturbing and accurate representation of the dehumanizing practices that were common in 1960s psychiatric hospitals. However, the ban forced the public to accept and adopt the perspective of the individuals who labeled *Titicut Follies* as indecent and a violation of patient/inmate privacy rights. For 21st century readers, the ban of *Titicut Follies* and the lack of reform would be analogous to if the recordings of the murder of George Floyd Jr. by a police officer were banned because of privacy rights and concerns about the grotesqueness of the police officer’s actions. When information and art content is uncensored, there are opportunities for people to learn about political, social, and cultural issues. In the development of information or artwork related to mental health topics, future generations will need to be considerate of veracity, aspects of informed consent/assent, and maintenance of an individual’s dignity to avoid harm to psychological and physical wellbeing and legal ramifications. Nevertheless, censorship is not the solution as it dispossesses people from challenging and depicting social conventions and issues.

## References

[bibr1-00332941211061080] Ahmad A. R. (2019). The effect of media censorship on freedom. In International Conference on Accounting, Business, Economics and Politics, Erbil, IQ, April 10-11, 2019. Tishk International University. 10.23918/ICABEP2019p23

[bibr2-00332941211061080] Altindag A. Yanik M. Ucok A. Alptekin K. Ozkan M. (2006). Effects of an antistigma program on medical students’ attitudes towards people with schizophrenia. Psychiatry and Clinical Neurosciences, 60(3), 283–288. 10.1111/j.1440-1819.2006.01503.x 16732743

[bibr3-00332941211061080] Arendt F. Romer D. (2020). Problems posed by the werther effect as a ‘net effect’: A comment on recent scholarly work on the effects of 13 reasons why. The British Journal of Psychiatry, 217(6), 665–666. 10.1192/bjp.2019.197 31679540

[bibr4-00332941211061080] Bak-Coleman J. B. Kennedy I. Wack M. Beers A. Schafer J. S. Spiro E. S. Starbird K. West J. D. (2021). Combining interventions to reduce the spread of viral misinformation. SocArXiv. 10.31235/osf.io/4jtvm PMC9584817 35739250

[bibr5-00332941211061080] Bakir V. McStay A. (2018). Fake news and the economy of emotions: Problems, causes, solutions. Digital Journalism, 6(2), 154–175. 10.1080/21670811.2017.1345645

[bibr6-00332941211061080] Benz G. R. Branton M. (2005). Intervention [TV series]. A&E.

[bibr7-00332941211061080] Bridge J. A. Greenhouse J. B. Ruch D. Stevens J. Ackerman J. Sheftall A. H. Horowitz L. M. Kelleher K. J. Campo J. V. (2020). Association between the release of Netflix’s 13 reasons why and suicide rates in the United States: An interrupted time series analysis. Journal of the American Academy of Child and Adolescent Psychiatry, 59(2), 236–243. 10.1016/j.jaac.2019.04.020 31042568 PMC6817407

[bibr8-00332941211061080] Bushman B. J. Cantor J. (2003). Media ratings for violence and sex: Implications for policymakers and parents. American Psychologist, 58(2), 130–141. https://psycnet.apa.org/doi/10.1037/0003-066X.58.2.130 12747015 10.1037/0003-066x.58.2.130

[bibr9-00332941211061080] Carrara B. S. Fernandes R. H. H. Bobbili S. J. Ventura C. A. A. (2020). Health care providers and people with mental illness: An integrative review on anti-stigma interventions. International Journal of Social Psychiatry, 67(7), 840–853. https://doi.org/10.1177%2F0020764020985891 33380251 10.1177/0020764020985891

[bibr10-00332941211061080] Charles L. (2006). Borat [motion picture]. 20th Century Fox.

[bibr11-00332941211061080] Chase D. Grey B. Green R. Burgess M. Landress I. S. Winter T. Weiner M. (1999). The Sopranos [TV series]. Chase films; Brad Grey Television; HBO Entertainment.

[bibr12-00332941211061080] Chiang T. J. Posner R. A. (2006). Censorship versus freedom of expression in the arts. In V.A. Ginsburgh & D.Throsby (Eds.),. Handbook of the economics of art and culture, 309-335. 10.1016/S1574-0676(06.

[bibr13-00332941211061080] Christenson P. (1992). The effects of parental advisory labels on adolescent music preferences. Journal of Communication, 42(1), 106–113. https://psycnet.apa.org/doi/10.1111/j.1460-2466.1992.tb00772.x

[bibr14-00332941211061080] Cooper M. (2017). Turning ‘a nightmare of ghoulish obscenities’ into a ballet nightmare-of-ghoulish-obscenities-into-a-ballet-titicut-follies-the-ballet-james-sewell.html. Retrieved August 1, 2021 from https://www.nytimes.com/2017/03/30/arts/dance/turning-a-.

[bibr15-00332941211061080] Copeland W. E. (2020). One reason why not [Editorial]. Journal of the American Academy of Child and Adolescent Psychiatry, 59(2), 216–218. 10.1016/j.jaac.2019.09.025 31589907 PMC7216976

[bibr16-00332941211061080] Cruikshank E. C. Sevigny P. R. (2020). Reasons why not: A critical review of the television series 13 reasons why. Canadian Journal of Counselling and Psychotherapy, 54(4), 803–818. 10.47634/cjcp.v54i4.69046

[bibr17-00332941211061080] Deflem M. (2020). Popular culture and social control: The moral panic on music labeling. American Journal of Criminal Justice, 45(1), 2–24. 10.1007/s12103-019-09495-3

[bibr18-00332941211061080] Domaradzki J. (2021). The werther effect, the papageno effect or no effect? A literature review. International Journal of Environmental Research and Public Health, 18(5), 2396. 10.3390/ijerph18052396 33804527 PMC7967741

[bibr19-00332941211061080] Dunkel C. S. Hillard E. E. (2014). Blasphemy or art: What art should be censored and who wants to censor it? The Journal of Psychology, 148(1), 1-–21. 10.1080/00223980.2012.730563 24617268

[bibr20-00332941211061080] Durham R. Wilkinson P. (2020). Joker: How ‘entertaining’ films may affect public attitudes towards mental illness–psychiatry in movies. The British Journal of Psychiatry, 216(6), 307. 10.1192/bjp.2020.56

[bibr21-00332941211061080] Erickson B. (2021). Deinstitutionalization through optimism: The community mental health act of 1963. American Journal of Psychiatry Residents Journal, 16(4), 6–7. 10.1176/appi.ajp-rj.2021.160404 37089526 PMC10116376

[bibr22-00332941211061080] Ferguson C. J. (2019). 13 reasons why not: A methodological and meta-analytic review of evidence regarding suicide contagion by fictional media. Suicide and Life Threatening Behavior, 49(4), 1178–1186. 10.1111/sltb.12517 30318609

[bibr23-00332941211061080] Ferguson C. J. (2021). One less reason why: Viewing of suicide-themed fictional media is associated with lower depressive symptoms in youth. Mass Communication and Society, 24(1), 85–105. 10.1080/15205436.2020.1756335

[bibr24-00332941211061080] Forman M. (1975). One flew over the cuckoo’s nest [motion picture]. United Artists.

[bibr25-00332941211061080] Godfrey P. C. (2007). Law and the regulation of the obscene. In Seidman S. Fischer N. Meeks C. (Eds.), Handbook of the new sexuality studies (pp. 372–380). Routledge. 10.4324/9780203963081-56

[bibr26-00332941211061080] Goffman E. (1961). Asylums: Essays on the social situation of mental patients and other inmates. Penguin 10.1192/bjp.bp.113.140442 25587580

[bibr27-00332941211061080] Gomez S. Matyka M. Son D. McCarthy T. Wettels J. G. Golin S. Sugar M. Teefey M. Laiblin K. (2017). 13 reasons why [TV series]. Netflix.

[bibr28-00332941211061080] Goodman W. (1993). Review/television; an unhealthy hospital stars in ‘titicut follies’. Retrieved August 3, 2021 from https://www.nytimes.com/1993/04/06/movies/review-television-an-unhealthy-hospital-stars-in-titicut-follies.html

[bibr29-00332941211061080] Gosselt J. F. Jong De M. D. Hoof Van J. J. (2012). Effects of media ratings on children and adolescents: A litmus test of the forbidden fruit effect. Journal of Communication, 62(6), 1084–1101. 10.1111/j.1460-2466.2011.01597.x

[bibr30-00332941211061080] Grant B. K. (1998). Ethnography in the first person. In Grant B. K. Sloniowski J. (Eds.), Documenting the documentary (pp. 238–253). Wayne State University Press.

[bibr31-00332941211061080] Griffith D. W. (1915). The birth of a nation [motion picture]. Triangle Film Corporation.

[bibr32-00332941211061080] Hippler F. (1940). The eternal Jew [motion picture]. Terra Film.

[bibr33-00332941211061080] Jones P. J. Bellet B. W. McNally R. J. (2020). Helping or harming? The effect of trigger warnings on individuals with trauma histories. Clinical Psychological Science, 8(5), 905–917. https://doi.org/10.1177%2F2167702620921341

[bibr34-00332941211061080] Kaufman A. (2011). Rehab television shows: Intervention or exploitation. L.A. Times . Retrieved July 27, 2021 from https://www.latimes.com/archives/la-xpm-2011-jan-02-la-ca-celebrity-rehab-20110102-story.html

[bibr35-00332941211061080] Kaufman M. R. Bazell A. T. Collaco A. Sedoc J. (2021). “This show hits really close to home on so many levels”: An analysis of reddit comments about HBO’s Euphoria to understand viewers’ experiences of and reactions to substance use and mental illness. Drug and Alcohol Dependence, 220, 108468. 10.1016/j.drugalcdep.2020.108468. 33540349 PMC8183393

[bibr36-00332941211061080] Kriegman J. Steinberg E. Despres E. B. (2019). Couples Therapy [TV series]. Showtime.

[bibr37-00332941211061080] Levinson B. (1988). Rain man [motion picture]. Metro-Goldwyn-Mayer Studios.

[bibr38-00332941211061080] Levinson S. Turen K. Nandan R. Drake G. “Future” Nur A. Leshem R. Levin D. Lichtenstein H. M. Lennon G. Toovi M. Yardeni T. Mokadi Y. Kleverweis J. Zendaya C. (2019) Euphoria [TV series]. HBO.

[bibr39-00332941211061080] Maliţa L. (2019). Arguing for art, debating censorship. Metacritic Journal for Comparative Studies and Theory, 5(1), 5–35. 10.24193/mjcst.2019.7.01

[bibr40-00332941211061080] Mangold J. (1999). Girl, interrupted [motion picture]. Columbia Pictures.

[bibr41-00332941211061080] McCann E. Huntley-Moore S. (2016). Madness in the movies: An evaluation of the use of cinema to explore mental health issues in nurse education. Nurse Education in Practice, 21, 37-43. 10.1016/j.nepr.2016.09.009. 27716595

[bibr42-00332941211061080] Miller v. California (1973). Miller v. California 413 U.S. 15.

[bibr43-00332941211061080] Moylan L. B. Needham I. McKenna K. Kimpel J. (2020). Location of power within psychiatry: A fifty-year journey as represented in film. Issues in Mental Health Nursing, 41(5), 378–384. 10.1080/01612840.2019.1675829 32186938

[bibr44-00332941211061080] Nevins S. (2007). Addiction [motion picture]. HBO.

[bibr45-00332941211061080] Niederkrotenthaler T. Stack S. Till B. Sinyor M. Pirkis J. Garcia D. Rockett I. R. H. Tran U. S. (2019). Association of increased youth suicides in the United States with the release of 13 reasons why. JAMA Psychiatry, 76(6), 933–940. 10.1001/jamapsychiatry.2019.0922 31141094 PMC6547137

[bibr46-00332941211061080] O’Rawe D. (2019). The politics of observation: Documentary film and radical psychiatry. Journal of Aesthetics & Culture, 11(1), 1568791. 10.1080/20004214.2019.1568791

[bibr47-00332941211061080] Pennycook G. Epstein Z. Mosleh M. Arechar A. A. Eckles D. Rand D. G. (2021). Shifting attention to accuracy can reduce misinformation online. Nature, 592(7855), 590–595. 10.1038/s41586-021-03344-2 33731933

[bibr48-00332941211061080] Pennycook G. Bear A. Collins E. T. Rand D. G. (2020a). The implied truth effect: Attaching warnings to a subset of fake news headlines increases perceived accuracy of headlines without warnings. Management Science, 66(11), 4944–4957. 10.1287/mnsc.2019.3478

[bibr49-00332941211061080] Pennycook G. McPhetres J. Zhang Y. Lu J. G. Rand D. G. (2020b). Fighting COVID-19 misinformation on social media: Experimental evidence for a scalable accuracy-nudge intervention. Psychological Science, 31(7), 770–780. https://doi.org/10.1177%2F0956797620939054 32603243 10.1177/0956797620939054 PMC7366427

[bibr50-00332941211061080] Pennycook G. Rand D. G. (2020). Who falls for fake news? The roles of bullshit receptivity, overclaiming, familiarity, and analytic thinking. Journal of Personality, 88(2), 185–200. 10.1111/jopy.12476 30929263

[bibr51-00332941211061080] Phillips T. (2019). Joker [motion picture]. Warner Bros.

[bibr52-00332941211061080] Pinsky D. Irwin J. (2008). Celebrity Rehab with Dr. Drew [TV series]. VH1.

[bibr53-00332941211061080] Sanson M. Strange D. Garry M. (2019). Trigger warnings are trivially helpful at reducing negative affect, intrusive thoughts, and avoidance. Clinical Psychological Science, 7(4), 778–793. https://doi.org/10.1177%2F2167702619827018

[bibr54-00332941211061080] Scalvini M. (2020). 13 reasons why: Can a tv show about suicide be ‘dangerous’? What are the moral obligations of a producer? Media, Culture & Society, 42(7–8), 1564–1574. https://doi.org/10.1177%2F0163443720932502

[bibr55-00332941211061080] Simon S. (2021). Trump’s insurrection and America’s year of living dangerously. Survival, 63(1), 7–16. 10.1080/00396338.2021.1881247

[bibr56-00332941211061080] Stewart E. G. (2020). United States law’s failure to appreciate art: How public art has been left out in the cold. Washington University Law Review, 97(4), 1233–1268

[bibr57-00332941211061080] Szasz T. (2007). The titicut follies: The forgotten story of a case of psychiatric censorship. History of Psychiatry, 18(1), 123–125. 10.1177/2F0957154X07073814 17580757

[bibr58-00332941211061080] Till B. Niederkrotenthaler T. Herberth A. Vitouch P. Sonneck G. (2010). Suicide in films: The impact of suicide portrayals on nonsuicidal viewers' well-being and the effectiveness of censorship. Suicide & Life-Threatening Behavior, 40(4), 319–327. 10.1521/suli.2010.40.4.319 20822358

[bibr59-00332941211061080] Williams A. R. Caplan A. L. (2012). Thomas Szasz: Rebel with a questionable cause. The Lancet, 381(9862), 1378–1379. 10.1016/S0140-6736(12 23091833

[bibr60-00332941211061080] Wiseman F. (1967). Titicut follies [motion picture]. Zipporah Films, Inc.

[bibr61-00332941211061080] Woliner J. (2020). Borat subsequent moviefilm [motion picture]. Amazon Studios.

[bibr62-00332941211061080] Yan J. (2017). Art in the dichotomy of freedom of expression & obscenity: An anti-censorship perspective. Manitoba Law Journal, 40(3), 365–390

[bibr63-00332941211061080] Yeung J. (2020). Kazakhstan embraces Borat catchphrase in new tourism campaign: 'Very nice!’. Retrieved July 25, 2021 from https://www.cnn.com/travel/article/kazakhstan-tourism-campaign-borat-intl-hnk-scli/index.html

